# Evaluation of the vascular protective effects of new oral anticoagulants in high-risk patients with atrial fibrillation (PREFER-AF): study protocol for a randomized controlled trial

**DOI:** 10.1186/s13063-016-1541-8

**Published:** 2016-08-24

**Authors:** Jin-Bae Kim, Hyun Jun Joung, Jung Myung Lee, Jong Shin Woo, Woo-shik Kim, Kwon Sam Kim, Kyung Hye Lee, Weon Kim

**Affiliations:** Division of Cardiology, Department of Internal Medicine, Kyung Hee University Hospital, Kyung Hee University, Seoul, Hoegi-dong 1, Dongdaemun-gu Seoul, 130-701 Republic of Korea

**Keywords:** Atrial fibrillation, Anticoagulant, Atherosclerosis, Endothelium

## Abstract

**Background:**

Atrial fibrillation (AF) is known to be associated with several pathophysiological mechanisms including endothelial dysfunction of the heart and arterial vessels. Recent evidence suggests that new oral anticoagulant (NOAC) treatment may improve endothelial function and the inflammatory process involved in atherosclerosis in AF patients. This study is designed to determine the efficacy of NOAC therapy in the prevention of endothelial dysfunction and the progression of atherosclerosis of AF subjects.

**Method/design:**

AF patients with a CHA2DS2-VASc score >2 and no previous history of overt coronary disease, severe peripheral arterial disease (PAD) or major stroke will be registered and randomly assigned either to the NOAC group (dabigatran or rivaroxaban) or the warfarin group in this prospective, randomized, 2-year follow-up study. Reactive hyperemia peripheral arterial tonometry (RH-PAT) measurements reflecting endothelial function will be conducted using the Endo-PAT2000 device. Left and right carotid intima-media thickness (IMT) will be measured at baseline, 12 months, and 24 months. The primary endpoint is defined as change in Reactive Hyperemia Index (RHI) at 12 months. Secondary endpoints included changes in the right and left maximum IMT of the common carotid artery (CCA) and internal carotid artery (ICA), the mean IMT of the CCA and ICA at 24 months, and 24-month cardiovascular events including cardiac death, stroke, acute myocardial infarction (AMI), overall cause of death, withdrawal of drug, or bleeding events.

**Discussion:**

This is the first study to evaluate the efficacy of NOAC therapy for the prevention of endothelial dysfunction and progression of atherosclerosis in AF subjects. These findings are expected to expand the knowledge of NOAC pleotropic action in AF patients.

**Trial registration:**

ClinicalTrials.gov: NCT02544932, registered on 7 September 2015.

## Background

Factor Xa and thrombin are well-known components of the coagulation cascade and other biological and pathophysiological processes that are known targets for effective anticoagulation treatment [1]. Therefore, the properties of oral, direct inhibitors of factor Xa (e.g., rivaroxaban) and thrombin (e.g., dabigatran) have been examined for hemostasis and thromboembolism management.

Accumulating evidence suggests that factor Xa and thrombin are important modulators of other cellular signaling mechanisms through the activation of protease-activated receptor (PAR)-mediated signaling. Cross-talk between coagulation and inflammatory pathways via thrombin- or factor Xa-mediated PAR activation on the arterial vessel wall and heart, and the resulting contribution to atherosclerosis and atrial fibrillation (AF), have been well-documented [2]. The endothelium, platelets, pro-inflammatory cytokines and chemokines, and several serine proteases (e.g., tissue factor (TF), factor Xa, thrombin), via the activation of PARs, are major points in for the promotion of inflammation and leukocyte migration, which results in the initiation of atherosclerosis [1–4].

Preclinical studies have provided evidence for the effects of direct Xa or thrombin inhibition beyond anticoagulation, including anti-inflammatory and protective activities in atherosclerotic plaque development [5–8]. Evidence has demonstrated that direct thrombin inhibition impairs the formation and size of atherosclerotic plaques in addition to preventing the progression of endothelial injury-associated stenosis in an apolipoprotein E-deficient mouse model [5, 6]. Also, factor Xa inhibition was shown to ensure the reduction of restenosis after balloon angioplasty of atherosclerotic femoral arteries in rabbits [7].

However, questions remain regarding the treatment concentration and adverse effects of these drugs. Furthermore, there is little evidence of similar effects in patients with AF. Therefore, this study will evaluate the protective effects of NOAC with reactive hyperemia peripheral arterial tonometry (RH-PAT) measurements made using the Endo-PAT2000 device, reflecting endothelial function and intima-media thickness (IMT) of the common carotid artery (CCA), which is used as a surrogate endpoint of atherosclerosis. In this study, we plan to analyze the occurrence of cardiovascular events in AF patients who are at high risk of these.

## Method/design

### Study subjects and hypothesis

Rivaroxaban and dabigatran have demonstrated potential for the management of thromboembolic disorders such as AF or deep vein thrombosis with or without pulmonary thromboembolism [9, 10]. We therefore plan to initiate a prospective, randomized, 2-year follow-up study to clarify the efficacy of rivaroxaban and dabigatran in affecting change of endothelial function and progression of atherosclerosis in AF patients. The working hypothesis of this trial is that rivaroxaban and dabigatran are superior to standard vitamin K (vitK)-antagonists in the prevention of endothelial dysfunction, assessed by peripheral arterial tone (PAT) ratio at 12 months, and the progression of CCA-IMT at 12-month and 24-month follow-up.

### Study design

**Fig. 1:**
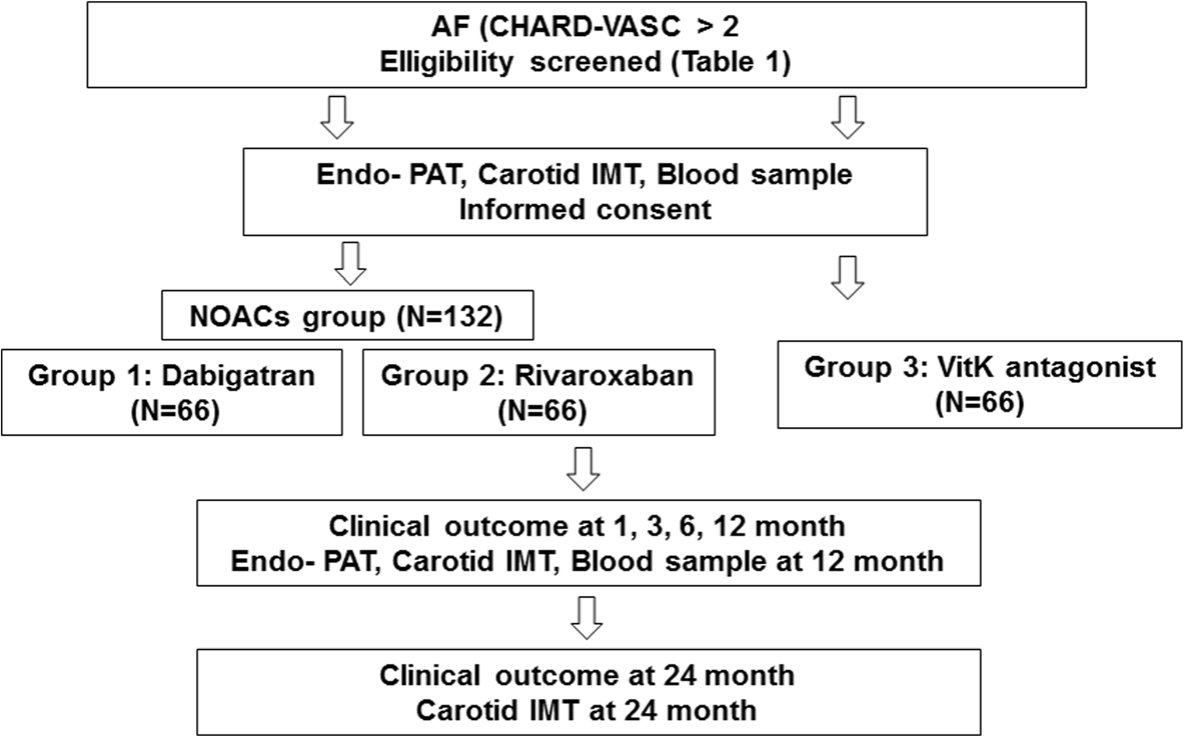
PREFER-AF trial flow diagram. Participants will receive new oral anticoagulant (NOAC) or vitamin K (vitK)-antagonist treatment according to the assigned sequence; then, endothelial function and clinical events will be assessed

This study is a prospective, randomized, 2-year follow-up study to clarify the efficacy of rivaroxaban and dabigatran in change of endothelial function and progression of atherosclerosis in AF patients. The study algorithm is shown in Fig. 1. After enrollment, subjects will be randomly assigned to the dabigatran group (110 mg or 150 mg twice/day; group 1) or the rivaroxaban group (20 mg/day; group 2) or the warfarin group (controlled by international normalized ratio (INR) of 2–3; group 3). After participation in the study procedure, clinical follow-up will occur at 1, 3, 12, and 24 months. Follow-up will be conducted via telephone interviews or office visits. Any adverse reactions will be assessed. This study is an investigator-initiated clinical trial with grant support from the Ministry of Health, Welfare, and Family Affairs of the Republic of Korea. The authors are solely responsible for the design, conduct, analysis, and manuscript preparation for this study. The 2-year duration of the study period will have a registration period from September 2015 to February 2016, with complete study duration from September 2015 to April 2018.

### Study population

Patients aged between 40 to 85 years will be enrolled in the study if they have been diagnosed with AF and have a CHA_2_DS_2_-VASc score above 2 points (Table 1). The following exclusion criteria have been set: severe peripheral arterial disease (PAD) (greater than a Fontaine IIb category), grade-4 or higher cerebral infarction on the modified Rankin Scale, and proven coronary artery disease based on coronary angiogram. In addition, patients with a range of concomitant comorbidities including severe hepatic or renal dysfunction, uncontrolled congestive heart failure, uncontrolled hypertension or diabetes mellitus, hematological disorders, or those with allergy or hypersensitivity to the investigational drugs, pregnant or lactating women, or women wishing to become pregnant, will be excluded. Written informed consent will be obtained from all patients, and the Ethics Review Board of Kyung Hee University Hospital will review the study.

**Table 1:** Enrollment criteria

Inclusion criteria	
1. AF (CHA_2_DS_2_-VASc score >2 points)	
2. Aged between 40 and 85 years	
Exclusion criteria	
1. Aged below 40 or over 85 years	
2. History of myocardial infarction	
3. Proven coronary artery disease by coronary angiogram	
4. Cerebrovascular disorders rated as grade 4 or greater on the modified Rankin Scale	
5. Severe peripheral arterial disease rated as Fontaine IIb or over	
6. Uncontrolled hypertension (180/110 mmHg) or diabetes (glycated hemoglobin: HbA1C >10.0 %)	
7. Severe hepatic dysfunction (aspartate transaminase (AST)/alanine transaminase (ALT) >100 mg/dL)	
8. Renal dysfunction (serum creatinine ≥2.0 mg/dL)	
9. Uncontrolled congestive heart failure	
10. Severe hematological abnormalities	
11. Drug allergies or history of hypersensitivity to the investigational drugs	
12. Pregnant or lactating women, or women who wish to become pregnant	

### Randomization and allocation concealment

Randomization will be performed using a computer-generated random number list with a permuted block design at a 1:1:1 ratio. Adequate allocation blinding will be ensured by the following procedure. After obtaining participant consent, allocation information will be sent to the main investigator at Kyung Hee University hospital by telephone, and allocations will not be known by the participants or enrolling investigators.

### Outcome measures

The primary endpoint is defined as change in the Reactive Hyperemia Index (RHI) at 12 months. Secondary endpoints included changes in the right and left maximum IMT of the CCA and internal carotid artery (ICA), mean IMT of the CCA and ICA at 24 months, and 24-month cardiovascular events including cardiac death, stroke, acute myocardial infarction (AMI), overall cause of death, withdrawal of drug, or bleeding events.

### Procedures

Measurement of PAT ratio will be performed with the standard technique and device [11–14]. Endo-PAT measurements will be performed at baseline and at 12 months after randomization. The RH-PAT 2000 device (Itamar Medical, Caesarea, Israel) will be used for digital RH-PAT to evaluate endothelial function as previously described. The RH-PAT Index (RHI) reflects the extent of reactive hyperemia and is calculated as the ratio of the average amplitude of PAT signal over 1 min starting 1.5 min after cuff deflation (control arm, A; occluded arm, C) divided by the average amplitude of PAT signal at the 2.5-min time period before cuff inflation (baseline) (control arm, B; occluded arm, D). Thus:

$$ RHI = \left(C/D\right)/\left(A/B\right) \times \mathrm{baseline}\ \mathrm{correction}. $$

Impaired endothelial function is defined as a log RHI less than 0.6, and favorable endothelial function is defined as a log RHI more than or equal to 0.6.

Measurement of CCA-IMT will be performed with the standard technique and device [12, 15]. Imaging studies of the left and right carotid arteries will be performed by expert sonographers who are well-trained in the use of 10-MHz linear vascular probes (Vivid 7, GE Vingmed Ultrasound, Horten, Norway). Measurements will be performed at baseline, and at 12 months and 24 months after randomization. To avoid interobserver variability, the same investigator will examine each participant with the same equipment throughout all the visits. The IMT will be measured as the distance between two parallel echogenic lines corresponding to the blood-intima and media-adventitia interface on the posterior wall of the artery. Three IMT determinations will be performed at the site of the thickest point with a maximum CCA-IMT and two adjacent points (1 cm upstream and 1 cm downstream from this site). These three determinations will be averaged (mean CCA-IMT). The CCA is defined as the segment extending from 10 to 20 mm proximal to the tip of the common carotid bifurcation site. The ICA is defined as the segment 10 mm distal to the tip of the common carotid bifurcation site, and the bifurcation is defined as the segment between the CCA and the ICA. Carotid IMT will be measured using dedicated software (Intimascope, Media Cross Co., Tokyo, Japan) by an examiner blinded to all clinical information.

Blood samples will be obtained from subjects in the fasting state. The von Willebrand factor, interleukin (IL)-10, and adiponectin endothelial cell markers, the IL-6, tumor necrosis factor alpha (TNF-α), and high-sensitivity C-reactive protein (hs-CRP) inflammatory markers, the vascular adhesion molecule and ICAM-1 adhesion molecules, and the CD-40 ligand, P-selectin, and cathepsin-K platelet activation markers will be measured using standard methods.

### Sample size measure and statistics

This study is a prospective, pilot study for a proof of concept trial. In a previous studies, the RHI was 1.63 ± 0.28 in patients with permanent/persistent AF [13, 14]. We assume that NOAC will improve RHI values by an 8 % difference with no significant difference between the two different NOACs. The expected difference in RHI was driven from clinical significances and previous medical treatments in other study population [16–18]. To detect a statistically significant difference with a power of 80 % with a two-sided *α*-level of 0.05, a sample of 165 patients (55 patients for each group) will be required. Assuming dropout rates would be 20 %, the total sample size will be set at 198 subjects.

Statistical analyses will be carried out using the SPSS Statistics for Windows ver. 17.0 software package (SPSS Inc., Chicago, IL, USA). A two-sided *p* < 0.05 is considered significant. Continuous variables, presented as means ± standard deviations, will be evaluated for normal distribution and compared using the Student *t* test or analysis of variance test accordingly. The continuous parameters with a skewed distribution will be logarithmically transformed and categorical variables, presented as frequencies and percentages, will be compared using the chi-square test or Fisher’s exact test when appropriate. Repeated measures parameters over time between the groups will be determined by repeated measure analysis of variance with an autoregressive model or analysis of covariance adjusting for baseline values. The clinical event rates will be assessed using the Kaplan-Meier method and the log-rank test will be used to compare survival between the two groups and the Cox proportional hazards model will be used for multivariate analysis (forward stepwise) to investigate which of the parameters identified using univariate analysis are significantly associated with clinical events.

## Discussion

The aim of this study is to determine the efficacy of NOAC therapy in the prevention of endothelial dysfunction and progression of atherosclerosis in AF subjects. Recent evidence suggests that AF adversely affects endothelial function [11, 13, 14]. Further, conversion of AF to sinus rhythm results in improved flow-mediated vasodilatation (FMD) in patients with persistent AF [13, 14]. It is not known whether the vitK antagonists are effective in affecting endothelial function and atherosclerotic progression in AF patients. Preclinical evidence shows that NOACs are effective in reducing inflammatory cytokines and atherosclerotic cascades [5–8]; however, clinical data are lacking. We believe that the therapeutic effects resulting from aspirin or warfarin and NOACs, which have different mechanisms of action, have not been sufficiently investigated in AF patients. The results of this study are expected to broaden our understanding of NOAC mechanisms in humans and lead to the development of appropriate drug therapies for patients with AF and atherosclerosis. This is a very important objective because the incidence of AF and its common complications are rising at a staggering rate in many countries.

One of the study limitations is that the primary outcome in this study is not a hard endpoint for clinical outcome but a surrogate marker for endothelial dysfunction. Therefore, further study for clinical outcome of vascular events comparing NOACs and warfarin should follow after this trial. Furthermore, this study is not double-blind trial. To minimize the bias, the technician performing examination and analysis will be blind to the allocation of patients.

### Trial status

This study is ongoing. We are currently recruiting and enrolling subjects at the Kyung Hee University Medical Center.

## Abbreviations

AF: Atrial fibrillation
AMI: Acute myocardial infarction
CCA: Common carotid artery
FMD: Flow-mediated vasodilatation
ICA: Internal carotid artery
IMT: Intima-media thickness
IRB: Institutional Review Board
NOAC: New oral anticoagulants
PAR: Protease-activated receptor
PREFER-AF: PREvention oF thromboembolic events – European Registry in Atrial Fibrillation
RHI: Reactive Hyperemia Index
RH-PAT: Reactive hyperemia peripheral arterial tonometry
